# The circadian clock regulates inflammatory arthritis

**DOI:** 10.1096/fj.201600353R

**Published:** 2016-08-03

**Authors:** Laura E. Hand, Thomas W. Hopwood, Suzanna H. Dickson, Amy L. Walker, Andrew S. I. Loudon, David W. Ray, David A. Bechtold, Julie E. Gibbs

**Affiliations:** Faculty of Biology, Medicine and Health, University of Manchester and Manchester Academic Health Sciences Centre, Manchester, United Kingdom

**Keywords:** rheumatoid arthritis, fibroblast-like synoviocyte, diurnal, cryptochrome

## Abstract

There is strong diurnal variation in the symptoms and severity of chronic inflammatory diseases, such as rheumatoid arthritis. In addition, disruption of the circadian clock is an aggravating factor associated with a range of human inflammatory diseases. To investigate mechanistic links between the biological clock and pathways underlying inflammatory arthritis, mice were administered collagen (or saline as a control) to induce arthritis. The treatment provoked an inflammatory response within the limbs, which showed robust daily variation in paw swelling and inflammatory cytokine expression. Inflammatory markers were significantly repressed during the dark phase. Further work demonstrated an active molecular clock within the inflamed limbs and highlighted the resident inflammatory cells, fibroblast-like synoviocytes (FLSs), as a potential source of the rhythmic inflammatory signal. Exposure of mice to constant light disrupted the clock in peripheral tissues, causing loss of the nighttime repression of local inflammation. Finally, the results show that the core clock proteins cryptochrome (CRY) 1 and 2 repressed inflammation within the FLSs, and provide novel evidence that a CRY activator has anti-inflammatory properties in human cells. We conclude that under chronic inflammatory conditions, the clock actively represses inflammatory pathways during the dark phase. This interaction has exciting potential as a therapeutic avenue for treatment of inflammatory disease.—Hand, L. E., Hopwood, T. W., Dickson, S. H., Walker, A. L., Loudon, A. S. I., Ray, D. W., Bechtold, D. A., Gibbs, J. E. The circadian clock regulates inflammatory arthritis.

The circadian clock is an internal timing mechanism that enables organisms to anticipate rhythmic daily changes in the environment and adapt their physiology accordingly. At a molecular level, 24-h oscillations are created by a transcriptional–translational feedback loop (1). Within this loop, the positive transcriptional regulators CLOCK and BMAL drive the transcription of the negative regulators period (*per*) and cryptochrome (*cry*). The PER and CRY proteins dimerize and inhibit CLOCK/BMAL-mediated transcription. The PER/CRY complex is subsequently degraded, releasing this inhibitory effect and allowing a new cycle of transcription to begin. Immune system function and activity are strongly influenced by the circadian clock, under both homeostatic conditions and during inflammatory challenge (2). Much of this temporal control of immune responses is orchestrated by peripheral clocks—timers within individual cellular components of the immune system that confer rhythmicity to their functionality.

Macrophages (3, 4), natural killer cells (5), T lymphocytes (6), mast cells (7), and eosinophils (8) all harbor cell autonomous clocks and exhibit daily rhythms in activity. Consequently, immune responses to acute challenge shows temporal gating, wherein response magnitude and characteristics vary in accordance with the phase of the circadian clock. For example, we have shown that the intrinsic clock within peritoneal macrophages drives the response to acute endotoxic challenge (3). Similarly, response to acute bacterial infection is circadian regulated (9–11). As yet, little is known regarding the influence of the clock on immune mechanisms underlying chronic inflammatory disorders. Investigation of this area could unmask critical links between immune and timing systems, enabling access to unexplored therapeutic options.

Rheumatoid arthritis (RA) is a debilitating autoimmune condition, characterized by inflammation and swelling within the joints. Inflammation within the joint is driven both by resident synovial cells [fibroblast-like synoviocytes (FLSs)] and by infiltrating immune cells (including monocytes, macrophages and T cells). In addition to acute swelling and pain, this chronic inflammatory state leads to joint remodelling, causing severe disability. RA shows daily variations in symptom and sign severity (12). Clinical studies dating back to the 1960s document patients with RA exhibiting increased joint stiffness in the morning (13), and it is well established that immunologic disease markers associated with RA show time-of-day variation. In particular, IL6 has been identified as strongly rhythmic in patients with RA, with elevated levels in the morning (14, 15). However, despite this clear clinical evidence of rhythmic disease presentation, little is known about how the pathophysiology of RA is governed by the circadian clock.

In this study, we characterized the role of the circadian clock in a well-established model of inflammatory arthritis, murine collagen-induced arthritis (CIA). Paralleling the rhythmic disease in patients with RA, we demonstrated robust 24-h variation in disease markers within arthritic mice, and identified FLSs as a rhythmic effector cell within the joints. Moreover, by disrupting the clock *in vivo* (*via* environmental disruption and genetic targeting) and *in vitro* (*via* pharmacological manipulation and RNA interference), we identified a direct role for the circadian clock in driving disease expression.

## MATERIALS AND METHODS

### Animals

All animal procedures were performed in accordance with the United Kingdom Animals (Scientific Procedure) Act 1986. Unless otherwise stated, animals were housed in 12:12 h light:dark (LD) lighting conditions with *ad libitum* access to normal rodent chow. DBA/1 mice were purchased from Envigo (Huntingdon, United Kingdom). mPER2::luc reporter mice (16), *cry1^−/−^cry2^−/−^* (17) and *per2*^−/−^ (18) mice were bred in house.

### CIA

Male DBA/1 mice age 8–12 wk were immunized with 1 mg/ml bovine type II collagen (MD Bioscience, Zurich, Switzerland) emulsified with an equal volume of Complete Freund’s Adjuvant (CFA; MD). The emulsion (50 μl) was injected intradermally, under anesthesia in 2 sites just above the base of the tail. As a control, animals were injected with an emulsion of saline and CFA in equal volumes. Twenty-one days later, a booster (200 µl 1 mg/ml collagen in saline) was administered intraperitoneally to CIA animals. Immunization and boost were routinely administered at zeitgeber time (ZT)6. Disease incidence and severity and hind paw thickness were monitored from ∼18 d postimmunization (DPI). Disease severity was scored in each limb on a 4-point scale: *1*) one inflamed digit; *2*), two or more inflamed digits; *3*), swelling of the foot pad and minor ankylosis; and *4*), severe swelling of the foot pad and joint and severe ankylosis), and the sum was calculated.

### Constant light

Mice were group housed in light-tight cabinets. At lights on, the light intensity was recorded at 42–45 lm/ft^2^. After 2 wk in 12:12 h LD, the lights were altered to remain constantly on (LL, light:light). For CIA induction, immunization occurred 2 wk after mice had been housed in LL, with animals remaining in LL for the duration of the study. Littermate control mice were housed in LD.

### Serum analysis

Blood samples were collected from the tail vein and allowed to clot at room temperature for 10 min before centrifugation (10,000 *g*, 10 min, 4°C). (Blood sampling during the dark phase was performed under dim red light). Serum was collected and frozen (−80°C) for later analysis. Analysis for cytokines was performed with a BioPlex Pro Mouse T_h_17 panel A/Pro Mouse cytokine 23-plex or customized kits as per kit instructions (Bio-Rad, Hemel Hempstead, United Kingdom). Serum samples (10 µl) were diluted in sample diluent (45 µl), and 50 µl was loaded per well.

### RNA analysis

Hindlimbs were dissected 1 mm above the ankle joint; the skin was removed and the tissue snap frozen. Each frozen joint was ground up in liquid nitrogen and transferred to Trizol in a Lysing D matrix tube (MP Biomedicals, Santa Ana, CA, USA) for further homogenization with a FastPrep machine (MP Biomedicals). Other tissues were placed directly into Trizol and homogenized with the FastPrep machine, as previously described. RNA was extracted using standard methods and quantified with a nanodrop and after DNase treatment converted to cDNA using standard procedures (RNA to cDNA; Thermo Fisher Scientific, Waltham, MA, USA). Quantitative PCR (qPCR) analysis was performed on a Step One Real-Time PCR System (Thermo Fisher Scientific) using TaqMan primers and probes. Murine *cxcl5*, *clock*, *icam1*, *ifnγ*, *rev-erbα*, *il10*, *bmal2*, and *npas2* primer/probe sets were purchased as validated TaqMan gene expression assays (Thermo Fisher Scientific). All other murine genes were analyzed with the primer and probe sequences listed on Supplemental Table 1. Human forward and reverse primers were used with probes from a Universal Probe Library Set (Roche Diagnostics, Ltd., Burgess Hill, United Kingdom) (Supplemental Table 1.). Analysis was comparative, and β-actin (mouse) and 18S (human) served as housekeeping genes. SYBR Green primers (Thermo Fisher Scientific) were validated by generating a melt curve to ensure a single PCR product, and gel analysis was performed to check the product size. Experimental samples were normalized to control samples, as indicated in Results.

### Murine FLS cultures

FLSs were isolated from the joints of mice as per published methods (19). Skin was removed from the hindpaws, which were then cut 1 mm above the ankle joint and placed into HBSS. After they were washed in HBSS and 70% ethanol, the limbs were dissected between the toe and ankle joints. Dissected tissue was placed into DMEM [1% penicillin-streptomycin (Pen-Strep); 10% fetal bovine serum (FBS)] containing 10 mg/ml collagenase solution (from *Clostridium* histolyticum type IV; Sigma-Aldrich, Dorset, United Kingdom) and placed in a shaking incubator for 1.5 h at 37°C. The cells, and some tissue debris, were pelleted by centrifugation, resuspended in DMEM, and cultured for at least 3 passages before experimental use to obtain a purified population of FLSs. To assess clock activity, the cells were synchronized with dexamethasone (DEX, 100 nM) and aliquots of cells were collected every 4 h for RNA analysis, starting 20 h after synchronization. To stimulate cells, TNFα was used at a concentration of 10 ng/ml. The CRY activator (KL001; Merck-Millipore, San Diego, CA, USA) was used at 8 µM (in DMSO) and applied 1 h before TNFα stimulation. The cells were harvested 2 h after stimulation (unless stated otherwise), and RNA was extracted with RNeasy kits (Qiagen, Manchester, United Kingdom). Population purity was assessed by flow cytometry with antibodies against CD11b (macrophage marker) and CD90.2 (FLS marker) (19). Cells derived from mPER2::luc mice were monitored on the photon multiplier tube (PMT) system for temporal luciferase activity (3).

### Small interfering RNA knockdown

Murine FLSs were synchronized with DEX (100 nM) and transfected with small interfering (si)RNAs targeting *cry1*, *cry2*, or a nontargeting control (SmartPool: On-TargetPlus mouse siRNAs; Dharmacon, Lafayette, CO, USA), using DharmaFect 2 (Dharmacon), as per the manufacturer’s instructions. The following day, the cells were treated with vehicle or TNFα (10 ng/ml) for 2 h at the peak of *cry1* expression, before they were harvested, and RNA was extracted with RNeasy kits (Qiagen).

### Human FLSs

Passage 2 human FLSs were purchased from Sigma-Aldrich and subcultured in DMEM (1% Pen-Strep; 10% FBS) for 2 to 3 rounds. Clock activity and CRY activator studies were performed as above.

### Fluorescence-activated cell sorting for FLSs

Both hindlimbs from naive DBA/1 mice were harvested and dissected at either ZT0 or -12, and the resultant tissue was pooled for collagenase digestion (as described above). Collagenase was applied for 1 h at 37°C and the cells pelleted. Cells were stained with Live/Dead fixable blue stain (Thermo Fisher Scientific Life Sciences) before application of an Fc block (eBioscience, San Diego, CA, USA) for 20 min and then with antibodies against CD45 (AF700, clone 30-F11) CD90.2 (BV786, clone 30-H12). Stained cells were sorted (Influx cell sorter; BD Biosciences, Franklin Lakes, NJ, USA) and single, live FLSs (CD45^-^ CD90.2^+^) were collected into an Eppendorf tube (Hamburg, Germany) containing RLT lysis buffer (RNeasy Micro Kit). The number of collected cells averaged 4.7 ± 0.4 × 10^4^/animal. Lysed cells were stored at −80°C until RNA extraction.

### ELISA

Quantification of cytokine levels in cell supernatants was performed with DuoSet ELISA kits from R&D Systems (Minneapolis, MN, USA) as per instructions. Where cytokine levels were normalized to protein content, bicinchoninic acid assays were performed to quantify protein levels according to kit instructions (Sigma-Aldrich).

### Statistics

Data were analyzed with IBM SPSS, v20 (Armonk, NY, USA) and Prism, v6 (GraphPad, La Jolla, CA, USA). Values presented are means ± sem. Cosinor analysis was used to assess temporal expression of clock genes in FLSs.

## RESULTS

### Arthritic mice exhibit diurnal rhythms in disease severity and joint inflammation

**Figure 1.**
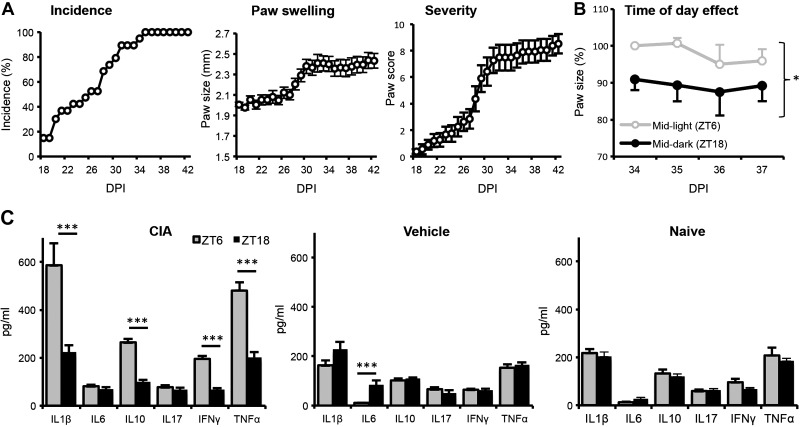
Diurnal variation in the disease pathology of CIA. *A*) Disease incidence, paw swelling, and disease severity scored by DPI in the murine CIA model (*n =* 19). *B*) Paw swelling of inflamed limbs (scoring 3 or 4) was measured in a subset of animals (*n =* 3 animals) at 8 consecutive times 12 h apart. For each inflamed paw, measurements were normalized to the first measurement at ZT6 (100%) (repeated-measures Student’s *t* test). *C*) Serum obtained from serial tail bleeds at ZT6 and -18 on DPI 30 from CIA (*n =* 7–8), vehicle (*n =* 4), or naive (*n =* 3–4) mice were analyzed for cytokine levels, 2-way ANOVA with *post hoc* Tukey. *P ≤ 0.05; ***P ≤ 0.005.

Collagen inoculation of DBA/1 mice resulted in a high incidence of inflammatory arthritis (CIA), with signs of disease evident from ∼18 DPI, peaking at ∼30–40 DPI (**Fig. 1*A***). In severely inflamed limbs showing swelling of the footpad (severity score, ≥3), paw size was significantly greater at mid-light phase (6 h after lights on; ZT6) when compared with the mid-dark phase (18 h after lights on; ZT18; Fig. 1*B*). Paw size did not vary in control naïve mice, or in the unaffected paws of CIA mice (data not shown). In line with day/night variation in paw swelling, CIA mice showed strong diurnal variation in levels of circulating cytokines (Fig. 1*C*). IL1β, IL10, IFNγ, and TNFα were significantly elevated at ZT6 compared with ZT18. ZT18 circulating inflammatory cytokines in the CIA mice were no higher than levels measured in matched naïve or vehicle-treated mice. In a separate experimental group, serum was sampled at ZT0 and -12 from arthritic and vehicle-treated mice and analyzed in a broad cytokine array (Supplemental Fig. 1). This confirmed that levels of IL1β, IL10, IFNγ, and TNFα (but not IL6) exhibit a strong diurnal variation in CIA mice and are heightened during the light phase. Additional cytokines (IL2, -3, -4, -5, -12p40, -12p70, and -13 and granulocyte-macrophage–colony stimulating factor) and chemokines (CCL2, -5, and -11) were also significantly higher in the serum at ZT0 *vs.* -12 in arthritic animals.

**Figure 2.**
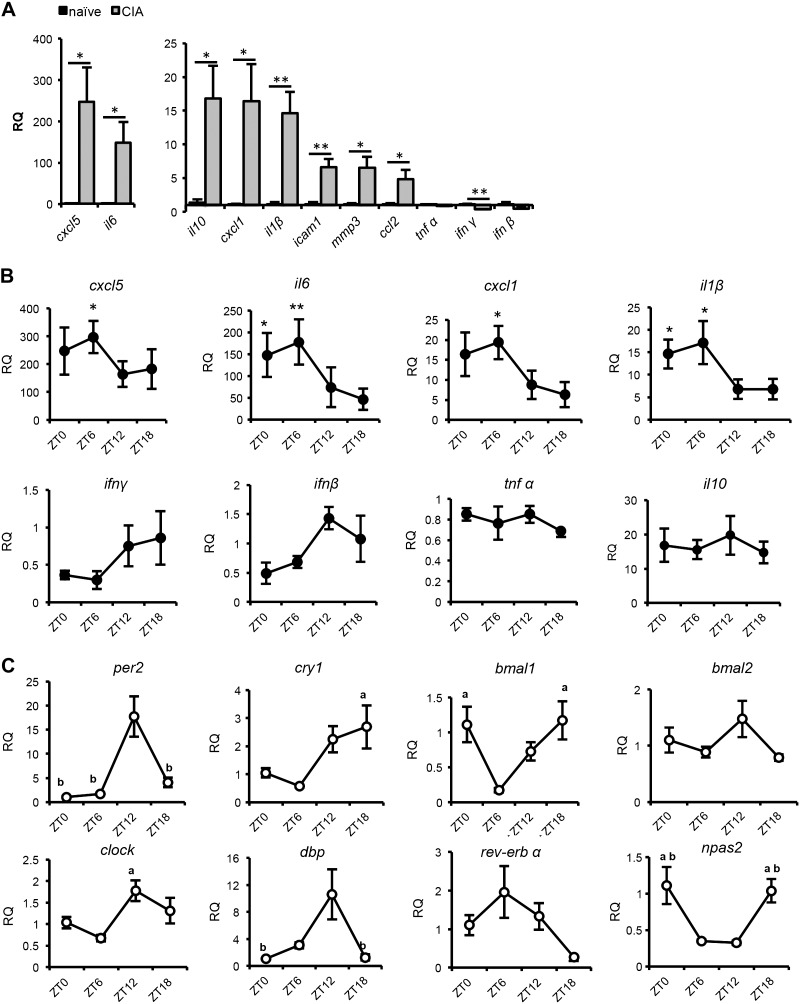
Expression of inflammatory markers and clock genes in inflamed limbs. *A*) Relative quantification of inflammatory gene transcripts in limbs harvested from naive (*n =* 4) or CIA (*n =* 5) animals at ZT0 (normalized to expression in naïve limbs; Student’s *t* test). *B*) Cytokine transcript levels were measured in limbs harvested from CIA mice at 4 time points across the day (*n =* 5). Values are made relative to samples collected from naive mice at ZT0. Kruskal-Wallis and *post hoc* Dunn comparing CIA values to naive values at ZT0. *C*) Clock gene transcript levels were measured in limbs harvested from CIA mice at 4 time points across the day (*n =* 5). Values are made relative to samples collected at ZT0. One-way ANOVA and *post hoc* Tukey. ^*a*^Significant change *vs.* ZT6. *^b^*Significant change *vs.* ZT12. *P ≤ 0.05; **P ≤ 0.01.

**Figure 3.**
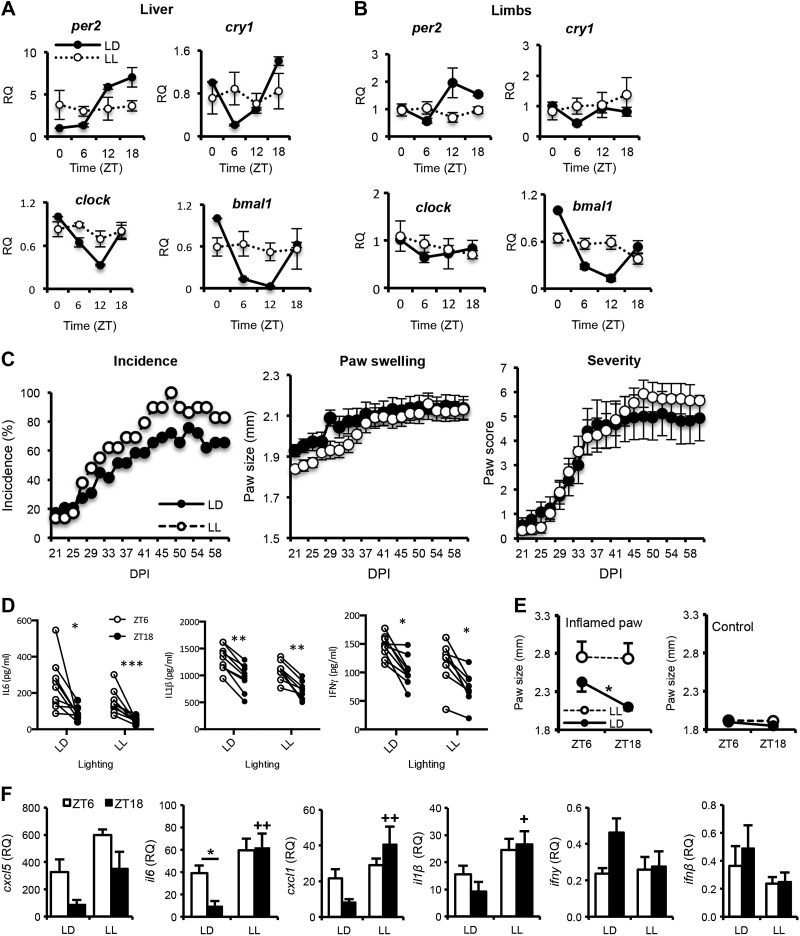
Circadian disruption abrogates rhythmic inflammation in the limbs. In these studies, samples were collected from LL and LD mice at the same time, with ZT in LD mice used as temporal markers. *A*, *B*) Control experiments confirmed that maintenance of animals in LL flattened circadian rhythms within peripheral organs (*A*, liver) and within the limbs (*B*), *n =* 3–4/time point. *C*) Housing mice in LL before and during CIA induction had no effect on the incidence, paw swelling or severity of CIA, *n =* 29/group. *D*) Serial blood samples taken from arthritic animals (*n =* 9/group) at 2 opposing time points 12 h apart (ZT6 and -18) were analyzed for cytokine levels with a BioPlex assay; paired samples are joined by lines (paired Student’s *t* test). *E*) In a separate cohort of animals, paw size was measured in severely inflamed paws (score 3–4, *n =* 4–5 animals) at 38 DPI (and in uninflamed paws as controls; *n =* 5–7 animals) at 2 time points 12 h apart. *F*) Cytokine transcript levels were analyzed in limbs harvested at ZT6 or -18 from CIA mice housed in LD or LL (*n =* 4–8) conditions; values were normalized to samples collected at ZT6 from nonarthritic mice housed in LD (2-way ANOVA and *post hoc* Tukey). *P ≤ 0.05, **P ≤ 0.01 for time-of-day differences (ZT6 *vs.* -18) within treatment groups. ^+^*P* ≤ 0.05, ^++^*P* ≤ 0.01 for significant lighting effect (LD *vs.* LL) at the same time point.

Having established a robust rhythmic inflammatory signal in the blood of CIA mice, we next addressed whether the inflamed joints themselves showed rhythmic changes in inflammatory state. Gene expression profiling of inflammatory markers in inflamed joint tissue of arthritic mice (collected at lights on; ZT0), revealed significantly increased expression of several proinflammatory (*cxcl5*, *il6*, *cxcl1*, *il1β*, and *ccl2*) and anti-inflammatory (*il10*) cytokines, as well as genes involved in cell adhesion (*icam1*) and tissue remodeling (*mmp3*) (**Fig. 2*A***). In line with previous reports (20), *tnfα* expression was unchanged and *ifnγ* and *ifnβ* showed significant reduction in expression levels. We next examined the temporal profile of this local inflammatory response across the 24-h cycle. Of the inflammatory cytokines up-regulated in CIA limbs, several exhibited rhythmic expression (Fig. 2*B*). *Cxcl5*, *il6*, *cxcl1,* and *il1β* levels were elevated during the light phase, whereas *ifnγ* and *ifnβ* transcription peaked in the dark phase, and *tnfα* and *il10* remained uniform across the cycle. In contrast, cytokine transcript levels (*cxcl5*, *il1β*, and *il6*) were flat throughout the day in limbs of naïve mice (Supplemental Fig. 2*A*). The data indicate that expression of inflammatory cytokines within the joint differs significantly from that of circulating profiles (*i.e.*, IL6, INFγ, TNFα), suggesting operation of a local, oscillating mechanism of cytokine expression within the joint. Temporal analysis of clock gene expression in inflamed limbs of CIA mice revealed robust 24 h rhythmicity of the clock genes (*per2*, *cry1*, *bmal1*, *clock*, *rev-erbα*, and *npas2*) and clock output genes (*dbp)* within this tissue (Fig. 2*C*), with a characteristic increase in the expression of *per2* and *cry1* (the negative arm of the clock) during the dark phase (ZT12–18). A similar pattern of clock gene expression was observed in healthy limbs from naive mice (**Fig. 3*B***), although the expression of most clock genes was reduced in the inflamed limbs (Supplemental Fig. 2*B*).

These studies reveal a time-dependent fluctuation in disease severity in CIA mice that correlates with daytime exacerbation of joint inflammation. Within the inflamed joint, the rhythmic proinflammatory signal (elevated *cxcl5*, *il6*, *cxcl1*, and *il1β*) occurs in antiphase to the negative arm of the clock (*per*, *cry*).

### Circadian disruption alters disease rhythmicity

Given the strong rhythmicity of disease activity in CIA mice, we examined the effects of constant light (LL exposure) on disease incidence, paw swelling, disease severity, and disease rhythmicity. Housing under LL disrupts rhythmic behavior in mice, and impairs peripheral clock oscillations (21, 22). This effect was confirmed in naive DBA/1 mice exposed to LL for 6 wk (Fig. 3*A*, *B*). Daily rhythms in peripheral (liver and limb) clock gene expression were profoundly decreased in LL-exposed mice in comparison to those in LD-housed controls. For CIA experiments, mice were exposed to LL from 14 d before collagen immunization until the end of the experiment. As predicted from control studies, arthritic mice kept in constant light showed loss of diurnal variation in clock gene expression in inflamed limbs (Supplemental Fig. 2*C*). Disease incidence, disease score, and paw thickness of CIA mice were not significantly different between the lighting regimens (Fig. 3*C*). Moreover, levels of circulating cytokines maintained a diurnal variation in arthritic mice held under LL (Fig. 3*D*). However, LL-exposed animals showed no diurnal variation in the paw size of severely affected limbs (unlike LD-housed mice) with loss of the nighttime repression (Fig. 3*E*). In line with this result, analysis of cytokine expression within inflamed paws at 2 time points (projected ZT6 and -18) confirmed consistently high levels of the proinflammatory cytokines *cxcl5*, *il6*, *cxcl1*, and *il1β* and consistently low levels of *ifnγ* and *ifnβ* (Fig. 3*F*).

### FLSs are rhythmic inflammatory effector cells within the joint

**Figure 4.**
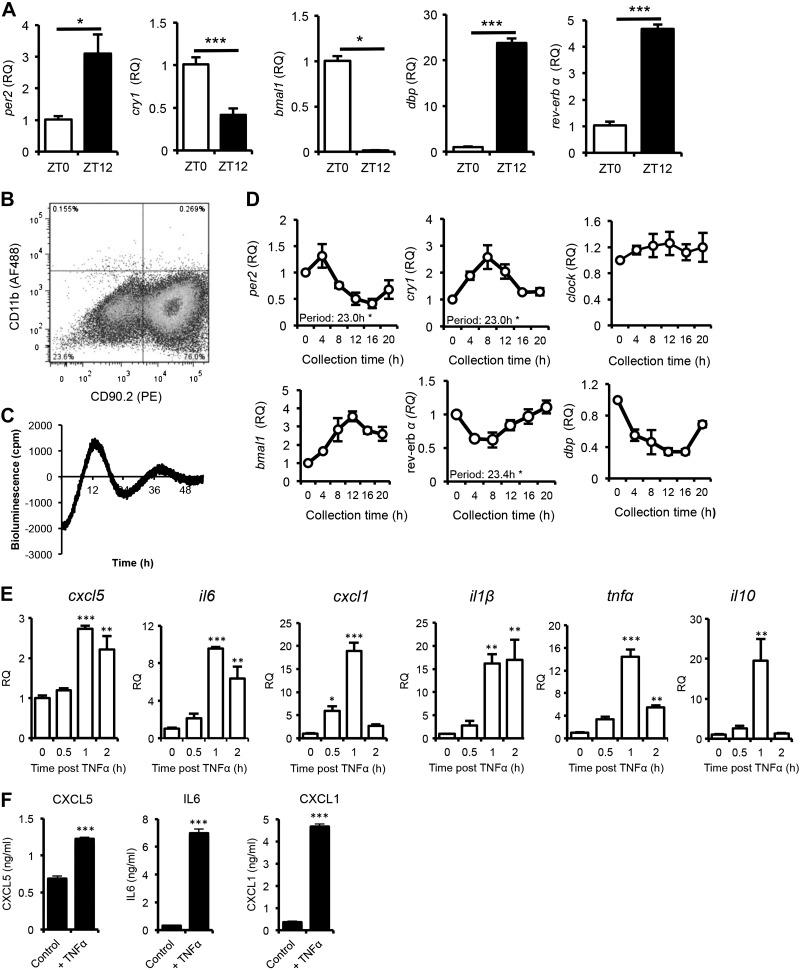
FLSs as rhythmic effector cells in the joints. *A*) FLSs sorted from naïve limbs of mice euthanized at ZT0 (*n =* 4) or ZT12 (*n =* 5) were analyzed for expression of clock genes. Clock gene expression was normalized to levels at ZT0, comparisons were made by using the Student’s *t* test. *B*) Flow cytometric analysis of expression of CD90.2 (FLS marker) and CD11b (macrophage marker) in synovial fibroblasts cultured from DBA/1 mice indicating population purity. *C*) FLSs isolated from PER2::luc mice showed rhythmic bioluminescent output under PMTs with a mean period of 24.8 ± 0.1 h (*n =* 6). *D*) FLSs cultured from DBA/1 mice (naive) showed circadian rhythmicity in core clock genes after synchronization (*n =* 3). *E*) Murine (DBA/1) synovial fibroblasts were challenged with TNFα for 30 min to 2 h and expression of inflammatory cytokines quantified relative to naive controls (*n =* 3); *ifnγ* and *ifnβ* levels were undetectable (1-way ANOVA and *post hoc* Bonferroni test). *F*) Murine (DBA/1) synovial fibroblasts were challenged with TNFα for 24 h. Cell supernatants were analyzed for cytokine production, Student’s *t* tests (*n =* 3); IL1β and IL10 levels were undetectable. *P ≤ 0.05; **P ≤ 0.01; ***P ≤ 0.005.

An earlier study has implicated FLSs as local mediators of inflammation in human RA and animal models (23). To assess the circadian rhythmicity of this cell population *in vivo*, FLSs were isolated (by FACS) from the limbs of naive DBA/1 mice at ZT0 and -12, and analyzed for expression of clock genes (**Fig. 4*A***). These samples showed significant diurnal oscillations in clock genes that followed the expected phase. To determine whether this rhythmicity continues *ex vivo* FLSs were extracted and cultured from naïve DBA/1 mice (population purity, 70–80%; Fig. 4*B*), as well as mPER2::luc clock reporter mice, which have a luciferase reporter fused to the PER2 protein (16). Real-time recording of mPER2::luc bioluminescence demonstrated that joint-derived FLSs exhibited an intrinsic rhythmicity in PER2 protein expression (Fig. 4*C*), indicating the operation of an intrinsic cellular clock. Similarly, FLSs cultured from naive DBA/1 mice, showed clear rhythmicity in the expression of core clock genes (Fig. 4*D*). As expected, FLSs showed a proinflammatory phenotype in culture in response to TNFα stimulation (Fig. 4*E*). TNFα administration caused a pronounced and rapid increase in *cxcl5*, *il6*, *cxcl1*, *il1β*, and *tnfα* gene expression, as well as a significant increase in CXCL5, IL6, and CXCL1 protein secretion into the culture medium (Fig. 4F). *Ifnγ* and *ifnβ* expression, as well as IFNγ, IL10, and IL1β remained below the limits of detection, even after stimulation (TNFα production was not measured because of the presence of recombinant mouse TNFα in the medium). Together, these results implicate FLSs as mediators of rhythmic inflammatory responses within the joint.

### Targeting the clock in RA

**Figure 5.**
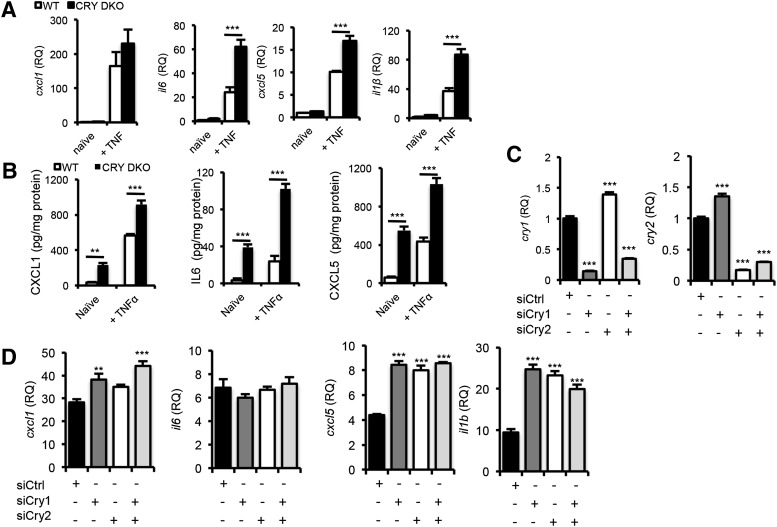
CRY represses inflammation. FLSs derived from CRY DKO mice or WT counterparts were stimulated with TNFα (10 ng/ml). *A*) Proinflammatory cytokine gene transcripts were quantified after 2 h relative to expression in naïve WT cells (2-way ANOVA and *post hoc* Bonferroni test). *B*) Protein levels in cell supernatants were quantified by ELISA after TNFα stimulation for 6 h, 2-way ANOVA, *post hoc* Bonferroni. *C*, *D*) FLSs derived from DBA/1 mice were transfected with siRNAs targeting *cry1* and *cry2*, or both, and stimulated with TNFα; qPCR was performed to confirm siRNA knockdown of target genes (*C*) and quantify proinflammatory cytokine gene transcripts (*D*). Data are relative to expression in control untreated cells, representative of 3 independent trials (1-way ANOVA and *post hoc* Bonferroni test). **P ≤ 0.01; ***P ≤ 0.005.

Our results show that arthritic joint inflammation is dampened during the night. Impairment of clock oscillations within the joint (through exposure to LL) causes loss of this nocturnal repression, leading to consistently active inflammation. We therefore tested whether nocturnal constraint on inflammatory processes involves the repressive arm of the molecular clock (which is most active at night) using FLSs harvested from *cry1^−/−^cry2^−/−^* [double knockout (DKO)] mice (**Fig. 5*A***, ***B***). Upon TNFα stimulation, DKO cells exhibited a significantly enhanced inflammatory response, at both the gene (*il6*, *cxcl5*, and *il1β*) and protein (CXCL1, IL6, and CXCL5) level. In contrast, FLSs derived from *per2^−/−^* mice (lacking the other component of the negative arm of the clock) showed no increase in inflammatory cytokine production in response to TNFα, compared to wild-type (WT) littermates (however, *cxcl5*/CXCL5 production was suppressed in the absence of PER2) (Supplemental Fig. 3). In further studies, WT FLS cells were treated with *cry1* or *cry2* siRNA, which resulted in 86 and 83% knockdown, respectively (Fig. 5*C*). Knockdown of *cry1*, *cry2*, or both significantly increased inflammatory cytokine production (*cxcl1*, *cxcl5*, and *il1β*) after TNFα stimulation (Fig. 5*D*).

**Figure 6.**
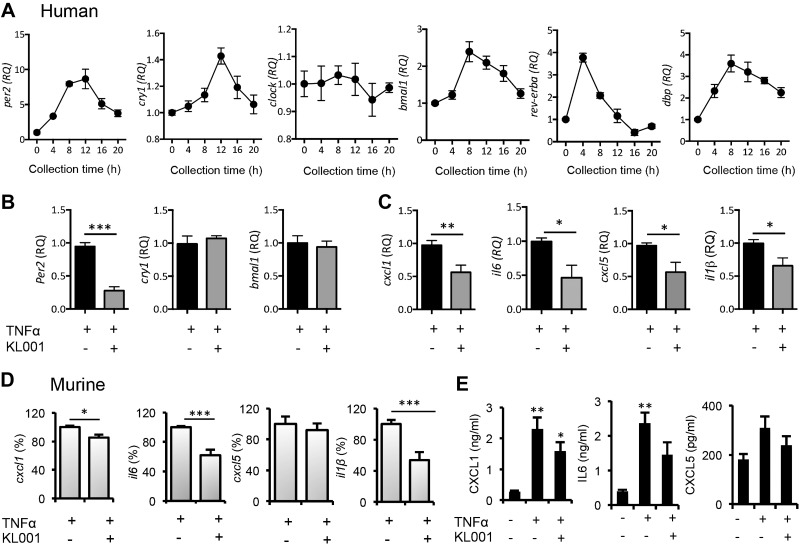
Anti-inflammatory properties of CRY activation on murine and human FLSs. *A*) Human FLSs were synchronized and then harvested every 4 h to assess rhythmic expression of core clock genes (*n =* 3). Human FLSs were stimulated with TNFα for 2 h with or without pretreatment for 1 h with 8 μM KL001. qPCR was performed to quantify clock gene expression (*B*) and proinflammatory cytokine gene transcripts (*C*). Data are relative to expression in control-treated cells, representative of 3 independent trials (Student’s *t* test). *D*) FLSs cells (passage 3) derived from DBA/1 mice were stimulated with TNFα for 2 h with or without pretreatment for 1 h with 8 μM KL001. qPCR was performed to quantify proinflammatory cytokine gene transcripts. Data are a percentage of cytokine transcription after TNFα stimulation to account for experimental variability between repeats (Student’s *t* test). *E*) CXCL1, IL6, and CXCL5 protein production by FLSs in response to TNFα stimulation for 4 h in the absence and presence of KL001 were quantified by Bioplex assay (*n =* 3); IL1β protein was not detectable (1-way ANOVA and *post hoc* Bonferroni test). *P ≤ 0.05; **P ≤ 0.01; ***P ≤ 0.005 *vs.* the control unstimulated group.

Finally, the CRY activator KL001 (24) (8 μM) was tested in murine and human FLSs to assess the anti-inflammatory effects of stabilizing the negative arm of the clock. FLSs obtained from human synovial tissue, demonstrated clear, robust rhythmicity of core clock genes after synchronization (**Fig. 6*A***). Control studies confirmed the anticipated effect of KL001 on the clock of FLSs. Expression of *per2* was rapidly suppressed (2 h); *cry1* and *bmal1* were not affected at this time point (Fig. 6*B*). Pretreatment with KL001 1 h before TNFα stimulation clearly repressed induction of *cxcl1/CXCL1*, *il6/IL6*, and *il1β* in both mouse and human cells (but only human *cxcl5*) (Fig. 6*C*–*E*), providing novel evidence that pharmacological stabilization of CRY has anti-inflammatory effects.

## DISCUSSION

The results of this study demonstrated diurnal variation in both the symptomatic expression of disease and the underlying inflammatory processes in a mouse model of inflammatory arthritis. We showed for the first time, the influence of circadian processes within the inflamed joint on disease severity and rhythmicity. Systemic and local profiling of proinflammatory cytokines and inflammatory markers revealed clear time-of-day variation, which showed significant elevations during the early part of the day (ZT0–6), compared to the night (ZT12–18), when signs of disease waned. Moreover, disruption of the circadian clock *in vivo* or in cultured FLSs, inflammatory effector cells within the joint, resulted in a heightened inflammatory state and loss of disease rhythmicity. The rhythmic disease profile in mice replicates observations in clinical studies (14, 15) where rhythmic pathophysiology is commonly observed in patients with RA. Rest–activity cycles of humans and mice are opposite in phase, thus time-of-day variation in inflammation is not simply related to sleep–wake cycles, but more likely to be an output of local clock activity.

Human RA patient studies consistently show elevated IL6 levels in the early morning (14, 15, 25). This is similarly reflected in the arthritic mice, with a morning peak in circulating cytokine levels. We now extend this observation to the local site of inflammation, the affected limb. Within the inflamed joint, daily rhythmicity in a wide range of proinflammatory markers (*cxcl5*, *il6*, *cxcl1*, and *il1β*) was observed, indicating that active proinflammatory processes in the joints of CIA mice are repressed during the night. The inverse relationship between IFNγ expression and footpad swelling has been reported in CIA mice (20, 26, 27). Although generally considered a proinflammatory cytokine, IFNγ has been assigned a protective role in the pathogenesis of arthritis, with genetic disruption of IFNγ resulting in increased disease activity. Similarly, *ifnβ* has anti-inflammatory properties in CIA (28). Both *ifnγ* and *ifnβ* levels were heightened during the dark phase. These data suggest that the clock exerts a synergistic effect on pro- and anti-inflammatory pathways to achieve overall repression of inflammation during the night, which parallels studies from Curtis *et al.* (29). We did not observe temporal variation in levels of the anti-inflammatory cytokine *il10* within the inflamed limb, which has been reported to be under clock control in peritoneal macrophages (29). This absence of variation could indicate that the cellular source of *il10* in the inflamed limb is not limited to the macrophage or may reflect differences in the nature of resident populations of macrophages. Differences between systemic and limb profiles of inflammatory markers were revealed (*e.g.*, IL6, TNFα, and IFNγ), indicating that a resident oscillator drives inflammation within the joint. Indeed, temporal analyses of clock gene expression in the joint demonstrated the presence of robust daily oscillations. There was clear rhythmicity in the expression of clock genes in inflamed limbs; however, overall we recorded a reduction in expression levels. This dampening could be due to the increased cellularity in inflamed limbs and an influx of nonrhythmic inflammatory cells into the tissue. Alternatively, this effect could be a consequence of the chronic inflammatory environment, as acute inflammation is known to have an impact on peripheral clock-dampening rhythms (30, 31).

Exposure of mice to constant light severely disrupts circadian rhythms in behavior and physiology and dampens rhythmicity in neuronal firing within the central clock, without affecting stress levels (21, 32–35). We therefore used LL as an environmental perturbation to examine how destabilization of the circadian clock impacts CIA disease incidence, severity, and rhythmicity. Our data showed the predicted dampening of rhythmicity in peripheral tissues under LL conditions in the liver and limbs in both naïve and arthritic conditions. Data regarding the impact of LL on peripheral rhythms was obtained by averaging data from multiple animals; thus, it is possible that the dampened rhythmicity observed reflects not only a decrease in amplitude within individual animals, but also a broadened distribution of circadian phase within the group (22). Given current knowledge of the detrimental impact of circadian disruption on innate immunity (36), it was somewhat surprising that mice held under LL conditions showed no significant difference in the incidence or overall severity of arthritic disease. However, disease severity (score and paw swelling) was routinely assessed at ZT6 (the peak of disease expression under LD), which may have minimized differences between LD- and LL-housed mice. We might predict that assessment at ZT18 would highlight differences between the lighting conditions. Furthermore, temporal variation in circulating levels of proinflammatory cytokines was maintained in LL-housed mice. In contrast, there was a clear impact of constant light on severely inflamed limbs (score, ≥3), wherein LL-housed mice exhibited an increase in magnitude and loss of diurnal rhythmicity in paw swelling. In line with this result, proinflammatory markers of severely affected limbs increased and were no longer rhythmic. Similarly, the nighttime increase in anti-inflammatory markers was abolished, clearly illustrating the importance of an intact local clock for generating a rhythmic inflammatory signature. It is of interest to define and quantify how cellular influx (*e.g.*, immune cell subtypes and number) into inflamed joints is altered by peripheral clock disruption.

Recently attention has turned to FLSs as critical players in the joint destruction related to arthritis. They become activated by the inflammatory milieu of arthritic joints, which alters their phenotype to a more proinflammatory, invasive type and causes epigenetic modifications that promote further aggressive features (37). In this study, murine FLSs are rhythmic *in vivo* and *ex vivo.* Similarly, in keeping with earlier studies, cultures of primary human FLSs exhibited robust temporal rhythms in clock gene expression (38, 39), thereby highlighting the FLSs as candidate drivers of rhythmic inflammation within arthritic joints. We demonstrated that these activated cells are a major source of the proinflammatory cytokines, which show rhythmic expression under chronic inflammatory conditions (CXCL1, CXCL5, and IL6). Within the inflamed joints, expression of *ifnγ* and *ifnβ* was in antiphase to the other proinflammatory cytokines examined. In addition, *ifnγ* and *ifnβ* transcript levels were not altered by application of TNFα to FLSs *in vitro*. This finding suggests that additional cell types within the limbs contribute to rhythmic cytokine signaling. Inflammatory cell infiltration of affected joints is pronounced in CIA. Therefore, migratory immune cells (*e.g.*, T cells, monocytes, neutrophils, and eosinophils) and other resident cells within the joint (*e.g.*, tissue macrophages, osteoclasts, osteoblasts, and osteocytes) may also contribute to the rhythmic patterns in joint inflammation during CIA. Nevertheless, it is established that the proinflammatory signature evoked by TNFα stimulation in FLSs differs significantly from that induced by other proinflammatory stimuli (40), and thus, within the inflamed joint, FLSs may still be a source of IFNγ/β. They showed rapid increases in transcription of *il1β* and *il10* in response to TNFα stimulation, but IL1β and IL10 protein was not detected in significant levels in cell supernatants, most likely reflecting the temporal dynamics of the protein production and sensitivity of our assays.

Taken together, our observations suggest that the repressive arm of the molecular clock, and specifically CRY, contributes to the reduction in inflammation observed during the night. Both CRY (41–44) and PER (45–48) have been implicated in regulation of inflammatory processes. However, although FLSs derived from mice lacking *cry1/2* demonstrated a proinflammatory phenotype, FLSs derived from *per2*^−/−^ mice did not show an increased response to TNFα stimulation. Loss of PER2 resulted in attenuated cxcl5/CXCL5 production. This supports our earlier work reporting circadian regulation of CXCL5 (9) and highlights the complex nature of the interactions between the molecular clock and inflammatory pathways. CRY has been linked to severity in an alternative model of arthritis, collagen antibody–induced arthritis. Mice lacking both *cry1* and *cry2* exhibit an exacerbation of joint swelling, compared to WT animals in this model caused by up-regulation of TNFα, IL-1β, and IL-6 (44). Although behaviorally arrhythmic in constant conditions, *cry1*^−/−^ *cry2*^−/−^ mice maintain robust diurnal patterns of activity when housed in a 12 h LD cycle, because of light-induced masking of activity (49). Thus, enhanced inflammation in *cry1*^−/−^ *cry2*^−/−^ mice can be attributed to loss of CRY *per se*, rather than loss in consolidated behavioral activity patterns. We showed that knockdown of both *cry* genes (*cry1^−/−^cry2^−/−^*) exacerbates inflammatory responses. siRNA knockdown studies revealed that both *cry1* and *cry2* are important for regulating inflammatory responses. There was no additional synergistic effect of *cry1* and *cry2* knockdown, suggesting that both genes play an important role. Given that genetic knockout of *cry1* and *cry2* caused a significant enhancement in the *il6* response to stimulation, it was surprising that siRNA knockdown had little effect, most likely reflecting our inability to achieve total knockdown through siRNA. Pharmacological activation of CRY proteins [with KL001 (24)] attenuates inflammatory responses in FLSs, in keeping with recent findings in a model of atherosclerosis, where overexpression of CRY1 had anti-inflammatory effects (41). This is the first demonstration of the anti-inflammatory properties of a synthetic CRY stabilizer. KL001 was originally developed as a tool to study CRY-dependent physiology and to facilitate development of clock-based therapeutics of diabetes (24). We now report the clock as a *bona fide* target for anti-inflammatory therapeutics in arthritis. Although the current formulations of CRY activators are not compatible with *in vivo* use, these findings highlight CRY activity as a novel avenue for therapeutic development in RA.

In summary, according to our data, local joint inflammation in CIA exhibits a robust diurnal variation, in keeping with observations in patients with RA. Furthermore, we identified an output pathway from the core clock to inflammation mediated by the CRY proteins, likely operating in FLSs within the affected joints. There has been a recent interest in the development of novel small molecule compounds that target components of the clock (*e.g.*, REV-ERB, ROR, and CRY) (24, 50–53), and these results open a potential new therapeutic avenue in the treatment of RA and other inflammatory conditions.

## Abbreviations

CFA: complete Freund’s adjuvant
CIA: collagen-induced arthritis
Cry: cryptochrome
Dex: dexamethasone
DKO: double knockout
DPI: days postimmunization
FBS: fetal bovine serum
FLSs: fibroblast-like synoviocytes
LD: light:dark
LL: light:light
Pen-Strep: penicillin-streptomycin
qPCR: quantitative PCR
RA: rheumatoid arthritis
siRNA: small interfering RNA
WT: wild-type
ZT: zeitgeber time

## References

[1] Hastings M. H., Maywood E. S., Reddy A. B. (2008) Two decades of circadian time. J. Neuroendocrinol. 20, 812–819 18601704 10.1111/j.1365-2826.2008.01715.x

[2] Labrecque N., Cermakian N. (2015) Circadian clocks in the immune system. J. Biol. Rhythms 30, 277–290 25900041 10.1177/0748730415577723

[3] Gibbs J. E., Blaikley J., Beesley S., Matthews L., Simpson K. D., Boyce S. H., Farrow S. N., Else K. J., Singh D., Ray D. W., Loudon A. S. (2012) The nuclear receptor REV-ERBα mediates circadian regulation of innate immunity through selective regulation of inflammatory cytokines. Proc. Natl. Acad. Sci. USA 109, 582–587 22184247 10.1073/pnas.1106750109 PMC3258648

[4] Keller M., Mazuch J., Abraham U., Eom G. D., Herzog E. D., Volk H. D., Kramer A., Maier B. (2009) A circadian clock in macrophages controls inflammatory immune responses. Proc. Natl. Acad. Sci. USA 106, 21407–21412 19955445 10.1073/pnas.0906361106 PMC2795539

[5] Arjona A., Sarkar D. K. (2005) Circadian oscillations of clock genes, cytolytic factors, and cytokines in rat NK cells. J. Immunol. 174, 7618–7624 15944262 10.4049/jimmunol.174.12.7618

[6] Bollinger T., Leutz A., Leliavski A., Skrum L., Kovac J., Bonacina L., Benedict C., Lange T., Westermann J., Oster H., Solbach W. (2011) Circadian clocks in mouse and human CD4+ T cells. PLoS One 6, e29801 22216357 10.1371/journal.pone.0029801 PMC3247291

[7] Wang X., Reece S. P., Van Scott M. R., Brown J. M. (2011) A circadian clock in murine bone marrow-derived mast cells modulates IgE-dependent activation in vitro. Brain Behav. Immun. 25, 127–134 20854894 10.1016/j.bbi.2010.09.007

[8] Baumann A., Gönnenwein S., Bischoff S. C., Sherman H., Chapnik N., Froy O., Lorentz A. (2013) The circadian clock is functional in eosinophils and mast cells. Immunology 140, 465–474 23876110 10.1111/imm.12157 PMC3839650

[9] Gibbs J., Ince L., Matthews L., Mei J., Bell T., Yang N., Saer B., Begley N., Poolman T., Pariollaud M., Farrow S., DeMayo F., Hussell T., Worthen G. S., Ray D., Loudon A. (2014) An epithelial circadian clock controls pulmonary inflammation and glucocorticoid action. Nat. Med. 20, 919–926 25064128 10.1038/nm.3599 PMC4268501

[10] Bellet M. M., Deriu E., Liu J. Z., Grimaldi B., Blaschitz C., Zeller M., Edwards R. A., Sahar S., Dandekar S., Baldi P., George M. D., Raffatellu M., Sassone-Corsi P. (2013) Circadian clock regulates the host response to Salmonella. Proc. Natl. Acad. Sci. USA 110, 9897–9902 23716692 10.1073/pnas.1120636110 PMC3683799

[11] Nguyen K. D., Fentress S. J., Qiu Y., Yun K., Cox J. S., Chawla A. (2013) Circadian gene Bmal1 regulates diurnal oscillations of Ly6C(hi) inflammatory monocytes. Science 341, 1483–1488 23970558 10.1126/science.1240636 PMC3836670

[12] Gibbs J. E., Ray D. W. (2013) The role of the circadian clock in rheumatoid arthritis. Arthritis Res. Ther. 15, 205 23427807 10.1186/ar4146 PMC3672712

[13] Ingpen M. L. (1968) The quantitative measurement of joint changes in rheumatoid arthritis. Ann. Phys. Med. 9, 322–327 4881234 10.1093/rheumatology/9.8.322

[14] Arvidson N. G., Gudbjörnsson B., Elfman L., Rydén A. C., Tötterman T. H., Hällgren R. (1994) Circadian rhythm of serum interleukin-6 in rheumatoid arthritis. Ann. Rheum. Dis. 53, 521–524 7944637 10.1136/ard.53.8.521 PMC1005392

[15] Perry M. G., Kirwan J. R., Jessop D. S., Hunt L. P. (2009) Overnight variations in cortisol, interleukin 6, tumour necrosis factor alpha and other cytokines in people with rheumatoid arthritis. Ann. Rheum. Dis. 68, 63–68 18375536 10.1136/ard.2007.086561 PMC2596304

[16] Yoo S. H., Yamazaki S., Lowrey P. L., Shimomura K., Ko C. H., Buhr E. D., Siepka S. M., Hong H. K., Oh W. J., Yoo O. J., Menaker M., Takahashi J. S. (2004) Period2:luciferase real-time reporting of circadian dynamics reveals persistent circadian oscillations in mouse peripheral tissues. Proc. Natl. Acad. Sci. USA 101, 5339–5346 14963227 10.1073/pnas.0308709101 PMC397382

[17] Sakhi K., Wegner S., Belle M. D., Howarth M., Delagrange P., Brown T. M., Piggins H. D. (2014) Intrinsic and extrinsic cues regulate the daily profile of mouse lateral habenula neuronal activity. J. Physiol. 592, 5025–5045 25194046 10.1113/jphysiol.2014.280065 PMC4259541

[18] Bae K., Weaver D. R. (2003) Light-induced phase shifts in mice lacking mPER1 or mPER2. J. Biol. Rhythms 18, 123–133 12693867 10.1177/0748730403252248

[19] Armaka M. G. V., Kontoyiannis D., Kolias G. (2009) A standardized protocol for the isolation and culture of normal and arthritogenic murine synovial fibroblasts. Nat. Protocol Exch. Accessed April 12, 2012, at: http://www.nature.com/protocolexchange/protocols/558

[20] Raatz Y., Ibrahim S., Feldmann M., Paleolog E. M. (2012) Gene expression profiling and functional analysis of angiogenic markers in murine collagen-induced arthritis. Arthritis Res. Ther. 14, R169 22817681 10.1186/ar3922 PMC3580563

[21] Deprés-Brummer P., Lévi F., Metzger G., Touitou Y. (1995) Light-induced suppression of the rat circadian system. Am. J. Physiol. 268, R1111–R1116 7771569 10.1152/ajpregu.1995.268.5.R1111

[22] Hamaguchi Y., Tahara Y., Hitosugi M., Shibata S. (2015) Impairment of circadian rhythms in peripheral clocks by constant light is partially reversed by scheduled feeding or exercise. J. Biol. Rhythms 30, 533–542 26467286 10.1177/0748730415609727

[23] Bartok B., Firestein G. S. (2010) Fibroblast-like synoviocytes: key effector cells in rheumatoid arthritis. Immunol. Rev. 233, 233–255 20193003 10.1111/j.0105-2896.2009.00859.x PMC2913689

[24] Hirota T., Lee J. W., St John P. C., Sawa M., Iwaisako K., Noguchi T., Pongsawakul P. Y., Sonntag T., Welsh D. K., Brenner D. A., Doyle F. J. III, Schultz P. G., Kay S. A. (2012) Identification of small molecule activators of cryptochrome. Science 337, 1094–1097 22798407 10.1126/science.1223710 PMC3589997

[25] Crofford L. J., Kalogeras K. T., Mastorakos G., Magiakou M. A., Wells J., Kanik K. S., Gold P. W., Chrousos G. P., Wilder R. L. (1997) Circadian relationships between interleukin (IL)-6 and hypothalamic-pituitary-adrenal axis hormones: failure of IL-6 to cause sustained hypercortisolism in patients with early untreated rheumatoid arthritis. J. Clin. Endocrinol. Metab. 82, 1279–1283 9100607 10.1210/jcem.82.4.3852

[26] Vermeire K., Heremans H., Vandeputte M., Huang S., Billiau A., Matthys P. (1997) Accelerated collagen-induced arthritis in IFN-gamma receptor-deficient mice. J. Immunol. 158, 5507–5513 9164974

[27] Williams A. S., Richards P. J., Thomas E., Carty S., Nowell M. A., Goodfellow R. M., Dent C. M., Williams B. D., Jones S. A., Topley N. (2007) Interferon-gamma protects against the development of structural damage in experimental arthritis by regulating polymorphonuclear neutrophil influx into diseased joints. Arthritis Rheum. 56, 2244–2254 17599735 10.1002/art.22732

[28] Van Holten J., Reedquist K., Sattonet-Roche P., Smeets T. J., Plater-Zyberk C., Vervoordeldonk M. J., Tak P. P. (2004) Treatment with recombinant interferon-beta reduces inflammation and slows cartilage destruction in the collagen-induced arthritis model of rheumatoid arthritis. Arthritis Res. Ther. 6, R239–R249 15142270 10.1186/ar1165 PMC416442

[29] Curtis A. M., Fagundes C. T., Yang G., Palsson-McDermott E. M., Wochal P., McGettrick A. F., Foley N. H., Early J. O., Chen L., Zhang H., Xue C., Geiger S. S., Hokamp K., Reilly M. P., Coogan A. N., Vigorito E., FitzGerald G. A., O’Neill L. A. (2015) Circadian control of innate immunity in macrophages by miR-155 targeting Bmal1. Proc. Natl. Acad. Sci. USA 112, 7231–7236 25995365 10.1073/pnas.1501327112 PMC4466714

[30] Haimovich B., Calvano J., Haimovich A. D., Calvano S. E., Coyle S. M., Lowry S. F. (2010) In vivo endotoxin synchronizes and suppresses clock gene expression in human peripheral blood leukocytes. Crit. Care Med. 38, 751–758 20081528 10.1097/CCM.0b013e3181cd131c PMC2929957

[31] Guo B., Yang N., Borysiewicz E., Dudek M., Williams J. L., Li J., Maywood E. S., Adamson A., Hastings M. H., Bateman J. F., White M. R., Boot-Handford R. P., Meng Q. J. (2015) Catabolic cytokines disrupt the circadian clock and the expression of clock-controlled genes in cartilage via an NFкB-dependent pathway. Osteoarthritis Cartilage 23, 1981–1988 10.1016/j.joca.2015.02.020 PMC4638193 26521744

[32] Coomans C. P., van den Berg S. A., Houben T., van Klinken J. B., van den Berg R., Pronk A. C., Havekes L. M., Romijn J. A., van Dijk K. W., Biermasz N. R., Meijer J. H. (2013) Detrimental effects of constant light exposure and high-fat diet on circadian energy metabolism and insulin sensitivity. FASEB J. 27, 1721–1732 23303208 10.1096/fj.12-210898

[33] Shi S. Q., Ansari T. S., McGuinness O. P., Wasserman D. H., Johnson C. H. (2013) Circadian disruption leads to insulin resistance and obesity. Curr. Biol. 23, 372–381 23434278 10.1016/j.cub.2013.01.048 PMC3595381

[34] Ohta H., Yamazaki S., McMahon D. G. (2005) Constant light desynchronizes mammalian clock neurons. Nat. Neurosci. 8, 267–269 15746913 10.1038/nn1395

[35] Alves-Simoes M., Coleman G., Canal M. M. (2016) Effects of type of light on mouse circadian behaviour and stress levels. Lab. Anim. 50, 21–29 10.1177/0023677215588052 25979911

[36] Castanon-Cervantes O., Wu M., Ehlen J. C., Paul K., Gamble K. L., Johnson R. L., Besing R. C., Menaker M., Gewirtz A. T., Davidson A. J. (2010) Dysregulation of inflammatory responses by chronic circadian disruption. J. Immunol. 185, 5796–5805 20944004 10.4049/jimmunol.1001026 PMC2974025

[37] Bottini N., Firestein G. S. (2013) Duality of fibroblast-like synoviocytes in RA: passive responders and imprinted aggressors. Nat. Rev. Rheumatol. 9, 24–33 23147896 10.1038/nrrheum.2012.190 PMC3970924

[38] Haas S., Straub R. H. (2012) Disruption of rhythms of molecular clocks in primary synovial fibroblasts of patients with osteoarthritis and rheumatoid arthritis, role of IL-1β/TNF. Arthritis Res. Ther. 14, R122 22621205 10.1186/ar3852 PMC3446503

[39] Kouri V. P., Olkkonen J., Kaivosoja E., Ainola M., Juhila J., Hovatta I., Konttinen Y. T., Mandelin J. (2013) Circadian timekeeping is disturbed in rheumatoid arthritis at molecular level. PLoS One 8, e54049 23335987 10.1371/journal.pone.0054049 PMC3546002

[40] Taberner M., Scott K. F., Weininger L., Mackay C. R., Rolph M. S. (2005) Overlapping gene expression profiles in rheumatoid fibroblast-like synoviocytes induced by the proinflammatory cytokines interleukin-1 beta and tumor necrosis factor. Inflamm. Res. 54, 10–16 15723199 10.1007/s00011-004-1315-8

[41] Yang L., Chu Y., Wang L., Wang Y., Zhao X., He W., Zhang P., Yang X., Liu X., Tian L., Li B., Dong S., Gao C. (2015) Overexpression of CRY1 protects against the development of atherosclerosis via the TLR/NF-κB pathway. Int. Immunopharmacol. 28, 525–530 26218278 10.1016/j.intimp.2015.07.001

[42] Qin B., Deng Y. (2015) Overexpression of circadian clock protein cryptochrome (CRY) 1 alleviates sleep deprivation-induced vascular inflammation in a mouse model. Immunol. Lett. 163, 76–83 25435215 10.1016/j.imlet.2014.11.014

[43] Narasimamurthy R., Hatori M., Nayak S. K., Liu F., Panda S., Verma I. M. (2012) Circadian clock protein cryptochrome regulates the expression of proinflammatory cytokines. Proc. Natl. Acad. Sci. USA 109, 12662–12667 22778400 10.1073/pnas.1209965109 PMC3411996

[44] Hashiramoto A., Yamane T., Tsumiyama K., Yoshida K., Komai K., Yamada H., Yamazaki F., Doi M., Okamura H., Shiozawa S. (2010) Mammalian clock gene Cryptochrome regulates arthritis via proinflammatory cytokine TNF-alpha. J. Immunol. 184, 1560–1565 20042581 10.4049/jimmunol.0903284

[45] Ando N., Nakamura Y., Aoki R., Ishimaru K., Ogawa H., Okumura K., Shibata S., Shimada S., Nakao A. (2015) Circadian gene clock regulates psoriasis-like skin inflammation in mice. J. Invest. Dermatol. 135, 3001–3008 26291684 10.1038/jid.2015.316 PMC4653315

[46] Xu H., Li H., Woo S. L., Kim S. M., Shende V. R., Neuendorff N., Guo X., Guo T., Qi T., Pei Y., Zhao Y., Hu X., Zhao J., Chen L., Chen L., Ji J. Y., Alaniz R. C., Earnest D. J., Wu C. (2014) Myeloid cell-specific disruption of Period1 and Period2 exacerbates diet-induced inflammation and insulin resistance. J. Biol. Chem. 289, 16374–16388 24770415 10.1074/jbc.M113.539601 PMC4047405

[47] Bonney S., Kominsky D., Brodsky K., Eltzschig H., Walker L., Eckle T. (2013) Cardiac Per2 functions as novel link between fatty acid metabolism and myocardial inflammation during ischemia and reperfusion injury of the heart. PLoS One 8, e71493 23977055 10.1371/journal.pone.0071493 PMC3748049

[48] Sugimoto T., Morioka N., Zhang F. F., Sato K., Abe H., Hisaoka-Nakashima K., Nakata Y. (2014) Clock gene Per1 regulates the production of CCL2 and interleukin-6 through p38, JNK1 and NF-κB activation in spinal astrocytes. Mol. Cell. Neurosci. 59, 37–46 24447840 10.1016/j.mcn.2014.01.003

[49] Van der Horst G. T., Muijtjens M., Kobayashi K., Takano R., Kanno S., Takao M., de Wit J., Verkerk A., Eker A. P., van Leenen D., Buijs R., Bootsma D., Hoeijmakers J. H., Yasui A. (1999) Mammalian Cry1 and Cry2 are essential for maintenance of circadian rhythms. Nature 398, 627–630 10217146 10.1038/19323

[50] Grant D., Yin L., Collins J. L., Parks D. J., Orband-Miller L. A., Wisely G. B., Joshi S., Lazar M. A., Willson T. M., Zuercher W. J. (2010) GSK4112, a small molecule chemical probe for the cell biology of the nuclear heme receptor Rev-erbα. ACS Chem. Biol. 5, 925–932 20677822 10.1021/cb100141y

[51] Solt L. A., Wang Y., Banerjee S., Hughes T., Kojetin D. J., Lundasen T., Shin Y., Liu J., Cameron M. D., Noel R., Yoo S. H., Takahashi J. S., Butler A. A., Kamenecka T. M., Burris T. P. (2012) Regulation of circadian behaviour and metabolism by synthetic REV-ERB agonists. Nature 485, 62–68 22460951 10.1038/nature11030 PMC3343186

[52] Kumar N., Solt L. A., Wang Y., Rogers P. M., Bhattacharyya G., Kamenecka T. M., Stayrook K. R., Crumbley C., Floyd Z. E., Gimble J. M., Griffin P. R., Burris T. P. (2010) Regulation of adipogenesis by natural and synthetic REV-ERB ligands. Endocrinology 151, 3015–3025 20427485 10.1210/en.2009-0800 PMC2903944

[53] Solt L. A., Kumar N., Nuhant P., Wang Y., Lauer J. L., Liu J., Istrate M. A., Kamenecka T. M., Roush W. R., Vidović D., Schürer S. C., Xu J., Wagoner G., Drew P. D., Griffin P. R., Burris T. P. (2011) Suppression of TH17 differentiation and autoimmunity by a synthetic ROR ligand. Nature 472, 491–494 21499262 10.1038/nature10075 PMC3148894

